# Effects and Mechanisms of Curcumin for the Prevention and Management of Cancers: An Updated Review

**DOI:** 10.3390/antiox11081481

**Published:** 2022-07-28

**Authors:** Zhi-Jun Yang, Si-Yu Huang, Dan-Dan Zhou, Ruo-Gu Xiong, Cai-Ning Zhao, Ai-Ping Fang, Yun-Jian Zhang, Hua-Bin Li, Hui-Lian Zhu

**Affiliations:** 1 Guangdong Provincial Key Laboratory of Food, Nutrition and Health, Department of Nutrition, School of Public Health, Sun Yat-Sen University, Guangzhou 510080, China; yangzhj57@mail2.sysu.edu.cn (Z.-J.Y.); huangsy9@mail2.sysu.edu.cn (S.-Y.H.); zhoudd6@mail2.sysu.edu.cn (D.-D.Z.); xiongrg@mail2.sysu.edu.cn (R.-G.X.); fangaip@mail.sysu.edu.cn (A.-P.F.); lihuabin@mail.sysu.edu.cn (H.-B.L.); 2 Department of Clinical Oncology, Li Ka Shing Faculty of Medicine, The University of Hong Kong, Hong Kong 999077, China; zhaocn@connect.hku.hk; 3 Department of Thyroid and Breast Surgery, The First Affiliated Hospital of Sun Yat-Sen University, Guangzhou 510080, China

**Keywords:** curcumin, anticancer, mechanism, bioavailability, safety

## Abstract

Cancer is the leading cause of death in the world. Curcumin is the main ingredient in turmeric (*Curcuma longa* L.), and is widely used in the food industry. It shows anticancer properties on different types of cancers, and the underlying mechanisms of action include inhibiting cell proliferation, suppressing invasion and migration, promoting cell apoptosis, inducing autophagy, decreasing cancer stemness, increasing reactive oxygen species production, reducing inflammation, triggering ferroptosis, regulating gut microbiota, and adjuvant therapy. In addition, the anticancer action of curcumin is demonstrated in clinical trials. Moreover, the poor water solubility and low bioavailability of curcumin can be improved by a variety of nanotechnologies, which will promote its clinical effects. Furthermore, although curcumin shows some adverse effects, such as diarrhea and nausea, it is generally safe and tolerable. This paper is an updated review of the prevention and management of cancers by curcumin with a special attention to its mechanisms of action.

## 1. Introduction

Cancer is the leading cause of death worldwide, with nearly 10 million deaths, and an estimated 19.3 million new cases in 2020, which is expected to reach 28.4 million new cases in 2040, an increase of 47% [1]. The cancer mortality burden is high in low- and middle-income countries [2]. At present, the most effective cancer therapies include immunotherapy, chemotherapy, radiotherapy and surgery. However, these therapeutic strategies have limited efficacies and potential side effects including fatigue, anorexia, liver and kidney damage, anxiety and depression, etc. [3,4,5,6]. On the other hand, some natural products, including fruits, vegetables, tea and spices have shown potential for the prevention and management of cancers, which have attracted wide attention from researchers [7,8,9,10,11,12,13,14,15,16].

Curcumin is extracted from the rhizome of turmeric (*Curcuma longa* L.), and is usually used as an aromatizer or a natural pigment in foods [17]. Curcumin possesses various biological activities, such as antibacterial, anti-inflammatory, antioxidant and anticancer effects [18,19,20,21,22,23]. Curcumin has shown anticancer effects on various cancers, such as breast, liver, lung, gastric and prostate cancers. For example, curcumin inhibited breast cancer MDA-MB-231 cells proliferation and induced apoptosis by increasing reactive oxygen species (ROS) production [24]. Curcumin also inhibited liver cancer HepG2 cells‘ proliferation, invasion and metastasis through inhibiting heat shock protein 70 (HSP70)- toll-like receptor 4 (TLR4) signaling [25]. Curcumin has been selected as a third-generation cancer chemopreventive agent by the National Cancer Institute [26]. This review paper summarizes the effects and mechanisms of curcumin on different cancers based on the results from cell and animal models as well as clinical trials published in the last five years, and special attention is paid to its mechanisms of action. In addition, several nanotechnologies are discussed to improve the bioavailability of curcumin. Finally, the adverse effects of curcumin are also highlighted. This paper will be helpful for the application of curcumin in the prevention and management of cancers.

## 2. Effects and Mechanisms of Curcumin on Cancers

**Figure 1:**
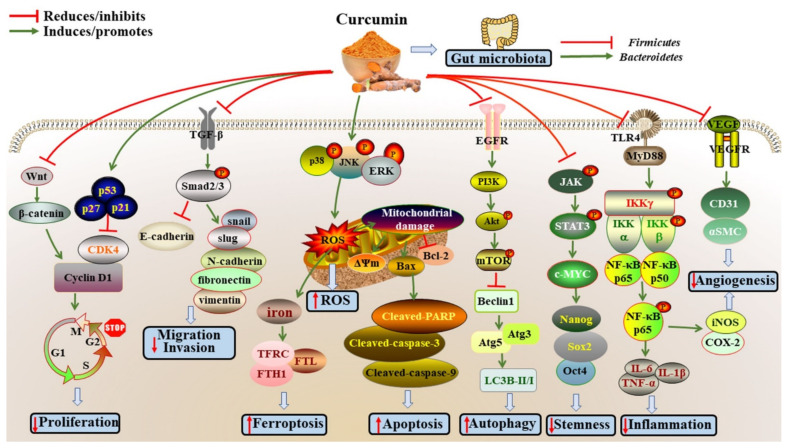
The main effects and mechanisms of curcumin on cancers. (1) Curcumin could suppress proliferation by attenuating cell cycle via inhibiting Wnt/β-catenin pathway, increasing the levels of p53, p21 and p27, and then inhibiting the levels of CDK4 and Cyclin D1. (2) Curcumin could enhance the levels of E-cadherin and decrease the levels of N-cadherin, vimentin, fibronectin, slug and snail through suppressing TGF-β/Smad2/3 pathway, ultimately inhibiting migration and invasion. (3) Curcumin could stimulate ROS production by activating p38 MAPK, JNK and ERK pathways. (4) Curcumin could trigger ferroptosis, and increase the levels of TFRC, FTL and FTH1. (5) Curcumin could promote apoptosis by enhancing the expression of apoptotic proteins (Bax, Cleaved-caspase-3, Cleaved-caspase-9 and Cleaved-PARP), and inhibiting the expression of anti-apoptotic proteins (Bcl-2). (6) Curcumin could enhance the expressions of Beclin1, Atg5, Atg3 and LC3B-II/I to promote autophagy by PI3K/Akt/mTOR pathway. (7) Curcumin could reduce the levels of Oct4, Sox2 and Nanog to suppress stemness through inhibiting JAK/STAT3 pathways. (8) Curcumin could suppress TLR4/NF-κB signaling pathway to attenuate inflammation (TNF-α, IL-6 and IL-1β). (9) Curcumin could attenuate angiogenesis by inhibiting the expressions of VEGF, CD31, αSMC, iNOS and COX-2. (10) Curcumin could regulate gut microbiota by reducing the ratio of Firmicutes/Bacteroidetes.

The anticancer effects of curcumin have been extensively studied in different cancers, such as breast, lung, colorectal, head and neck, gastric, bladder, prostate, thyroid, liver, ovarian, oral, pancreatic, cervical, tongue and brain cancers (Table 1 and Figure 1). The underlying mechanisms will be discussed in detail below.

Abbreviations: Akt, protein kinase B; Atg3, autophagy related 3; Atg5, autophagy related 5; Bax, Bcl-2 associated X protein; Bcl-2, B-cell lymphoma-2; Caspase-3, cysteinyl aspartate specific proteinase 3; Caspase-9, cysteinyl aspartate specific proteinase 9; CDK4, cyclin dependent kinase 4; EGFR, phospho-epidermal growth factor receptor; ERK, extracellular regulated protein kinases; FTH1, ferritin heavy chain 1; FTL, ferritin light chain; G1, where cells decide to grow and divide or enter the G0 phase (enter quiescence); G2, preparation for mitosis; JAK, Janus kinase; JNK, c-Jun N-terminal kinase; IKK, inhibitor of nuclear factor kappa-B kinase; IL-1β, interleukin-1β; IL-6, interleukin-6; IL-1β, interleukin-1β; LC3, microtubule-associated protein light chain 3; M, mitosis; mTOR, mammalian target of rapamycin; MyD88, myeloid differentiation primary response 88; NF-κB, nuclear factor kappa-B; Oct4, Octamer-binding transcription factor 4; p38 MAPK, p38 mitogen-activated protein kinase; PARP, poly (ADP-ribose) polymerase; PI3K, phosphatidylinositol-3-kinase; ROS, reactive oxygen species; S, DNA synthesis; Smad2/3, SMAD family member 2/3; Sox2, Sex determining region Y-box 2; TFRC, transferrin receptor; STAT, signal transducer and activator of transcription; TGF-β, transforming growth factor beta; TLR4, toll-like receptor 4; TNF-α, tumor necrosis factor α; VEGF, vascular endothelial growth factor. 

### 2.1. Inhibiting Cancer Cell Proliferation

Uncontrolled cell proliferation is a hallmark of cancer, and anti-proliferation is an important therapeutic intervention [95,96,97]. Many studies have found that curcumin could inhibit cancer cell proliferation. For example, a study showed that curcumin could reduce the viability of triple-negative breast cancer MDA-MB-231 and MDA-MB-468 cells, and it could also inhibit colony proliferation via inhibiting the Hedgehog pathway and the downstream target gene expression of PTCH1, SMO, Gli1 and Gli2 [27]. Furthermore, curcumin showed inhibition effects on the proliferation of prostate cancer PC-3 and DU145 cells through significantly increasing the expression of miR-34a [76]. Meanwhile, the cell cycle, a highly regulated process, is involved in enabling cell growth, cell division and duplication of genetic material [98]. Cyclin is often overactive in cancer cells, leading to uncontrolled proliferation of cancer cells, and targeting the cell cycle is considered as one of the targets of cancer therapy [99]. The cell cycle is composed of four phases: G1 (where cells decide to grow and divide or enter the G0 phase (enter quiescence)), S (DNA synthesis), G2 (preparation for mitosis), and M (mitosis) [100,101]. Cell cycle proteins are aberrantly activated in human cancers, which plays a pathogenic role in the development of most tumors [98]. A study found that curcumin could induce subG1 population accumulation and trigger G2/M arrest in breast cancer MCF-7, MDA-MB-453 and MDA-MB-231 cells, and upregulate the expression levels of p21 by targeting NF-κB signaling [36]. In addition, similar effects of curcumin on inducing G2 phase cell accumulation was observed in head and neck cancer SCC-9 cells, which indicated that curcumin could induce G2/M cell cycle arrest through inhibiting phosphatidylinositol-3-kinase (PI3K)/protein kinase B (Akt)/mammalian target of the rapamycin (mTOR) pathway [64].

Some in vivo studies have found that curcumin can inhibit tumor growth. For example, curcumin could reduce lung tumor volume and weight in the BALB/c nude mice xenograft model by inhibiting circ-PRKCA [38]. Moreover, curcumin suppressed ovarian cancer growth in xenograft models by up-regulating circ-PLEKHM3 [86]. Curcumin could also reduce a transformative phenotype and tumor formation in the 4-nitroquinoline-1-oxide-induced head and neck cancer model, and tumor volume was significantly reduced after curcumin treatment [63]. Another study found that curcumin significantly reduced tumor weight and tumor size in BALB/c nude mice with SGC-7901 gastric cancer cells’ subcutaneous xenografts by promoting miR-34a expression [68]. In addition, the liver tumor volume and weight were significantly decreased by curcumin in a HepG2 xenograft mouse model [79].

### 2.2. Inhibiting Invasion and Migration

Cancer cells have the ability to migrate and invade extensively, and cancer invasion and metastasis are landmark events in the transformation of locally grown tumors into systemic, metastatic, and life-threatening cancers [102,103]. Activation of the epithelial-mesenchymal transition (EMT) program may be a potential mechanism of cancer migration and invasion [104], conferring metastatic properties to cancer cells through raising invasiveness, mobility and resistance to apoptotic stimuli [105]. Inhibition of cancer cell migration and invasion may be one of the most essential anticancer mechanisms of curcumin. A study found that curcumin reduced breast cancer MCF-7 cell migration, as shown in the wound healing assay. At the same time, the results of the Transwell invasion assay also showed that curcumin significantly reduced MCF-7 cell invasion. The potential mechanisms might be attenuating lncRNA H19 [29]. Another study suggested that the migration and invasion of papillary thyroid cancer TPC-1 and BCPAP-R cells were suppressed by curcumin through up-regulation of miR-301a-3p [78]. Furthermore, curcumin significantly inhibited wound closure and invasion of pancreatic cancer Patu8988 and Panc-1 cells, which was mediated by inhibiting neural precursor cell expressed developmentally down-regulated protein 4 (NEDD4)/Akt/mTOR pathway [90]. Additionally, curcumin supplementation significantly reduced N-cadherin, twist, snail and vimentin, and increased E-cadherin in colorectal cancer SW480 cells, indicating that curcumin could suppress the EMT process by suppressing caudal type homeobox 2 (CDX2)/Wnt family member 3a (Wnt3a)/β-catenin pathway [55]. Moreover, curcumin decreased EMT of cervical cancer SiHa cells via pirin-dependent mechanism, enhanced the expression of E-cadherin and reduced the expression of N-cadherin, vimentin, slug and Zinc finger E-box binding homeobox 1 (Zeb1) through decreasing the levels of Pirin, which was further verified after Pirin knockdown [92].

### 2.3. Inducing Cell Apoptosis

Apoptosis is a kind of programmed cell death that occurs in an ordered and coordinated manner under pathological and physiological conditions and plays a crucial role in organism development and tissue homeostasis [106]. Apoptosis is associated with TNF-α, ROS and the activation of cysteine-protease and caspases [107]. During normal conditions, apoptosis is necessary for homeostasis but, in cancer, cells lose the ability to undergo apoptosis-induced death, leading to uncontrolled cell proliferation, which further leads to tumor survival, therapeutic resistance and cancer recurrence [108,109]. It was found that selectively inducing apoptosis in cancer cells has been considered as a promising treatment for many cancers [110]. A study found that the apoptotic ratios of breast cancer MDA-MB-231 and MDA-MB-468 cells were increased after treatment of curcumin, which was mediated by increasing the level of cysteinyl aspartate specific proteinase 9 (Caspase-9), and reducing the level of B-cell lymphoma-2 (Bcl-2) [32]. Another study pointed out that curcumin promoted prostate cancer PC-3 and DU145 cells apoptosis via enhancing the expression of miR-30a-5p and downregulating PCNA clamp associated factor (PCLAF) expression to increase the levels of Bcl-2 associated X protein (Bax) and Cleaved-cysteinyl aspartate specific proteinase 3 (Caspase-3), and to decrease the expression of Bcl-2 and Caspase-3 [73]. Furthermore, curcumin exerted a pro-apoptotic effect in cervical cancer Siha cells through increasing the expression levels of Cleaved-poly (ADP-ribose) polymerase (PARP) and Cleaved-caspase-3 [91]. Curcumin could also effectively promote the numbers of apoptotic tongue cancer CAL 27 cells, and decrease the expression of Bcl-2, increase the expressions of Bax and Cleaved-caspase-3 by regulating oxygen-related signaling pathways [93].

### 2.4. Inducing Autophagy

Autophagy is another kind of programmed cell death, which is essential for maintaining cellular homeostasis in stressful conditions [111]. Dysregulation of autophagy has implications in disease [111,112]. Enhanced autophagy could enhance anticancer immune responses, therefore targeting autophagy is a potential approach to improve the efficacy of current cancer treatments [113]. Curcumin-induced autophagy in cancers is one of the main concerns of many research projects. A study pointed out that curcumin could induce the formation of autophagic vesicle by suppressing AKT/mTOR/p70S6K pathway in ovarian cancer A2780 cells, and enhancing the expression of microtubule-associated protein light chain 3B I/II (LC3B-I/II), autophagy-related 3 (Atg3) and Beclin1 [85]. In another study, curcumin inhibited LC3I expression, and enhanced LC3II, Beclin1, Atg3 and autophagy related 5 (Atg5) expression in gastric cancer SGC-7901 and BGC-823 cells. The potential mechanisms might be inhibiting PI3K/Akt/mTOR pathway and activating P53 signaling pathway [69]. Meanwhile, curcumin was found to induce autophagy through suppressing PI3K/Akt/mTOR pathway, decreasing p62 expression, and increasing the expression of Beclin1 and LC3-II in lung cancer A549 cells [47]. Besides, curcumin could downregulate the expression of p62, and increase autolysosome and the expression of Beclin1 and LC3-II, thereby inducing autophagy [41].

### 2.5. Suppressing Cancer Cell Stemness

Cancer stem cells have self-renewal ability, which may lead to therapeutic resistance, tumor progression and relapse [114,115]. Cancer cell stemness refers to the stem cell-like phenotype of cancer cells [116]. Therefore, targeting cancer cell stemness may provide more specific treatments and exert better efficacy, and curcumin targeting cancer cell stemness has been shown to be one of the mechanisms of cancer treatment. CD44 and CD133 are well-known markers of cancer stem cells. In a study, curcumin supplementation significantly reduced the expression of CD44 and the number and size of tumor sphere formation of colon cancer HCT-116 and HCT-8 cells, which indicated that curcumin could inhibit the stem-cell like characteristics in colon cancer cells [62]. Moreover, curcumin could activate the Hippo pathway in lung cancer A549 and NCI-H1299 cells, and inhibit the expression of CD133, epithelial cell adhesion molecule (Epcam) and Octamer-binding transcription factor 4 (Oct4) [43]. Furthermore, curcumin significantly inhibited stem cell-like properties by reducing CD44^+^CD24^−^ cell subpopulation, the expression of Oct4, Nanog and Sex determining region Y-box 2 (Sox2) in breast cancer MCF-7 and MDA-MB-231 cells [33]. Meanwhile, another study found that curcumin inhibited the expression of Oct4 and Sox2 by suppressing Hedgehog/Gli1 pathways in breast cancer MDA-MB-231 and MDA-MB-468 cells [27].

### 2.6. Increasing ROS Production

ROS is inextricably linked to cancer progression and therapy, which may be associated with complex ROS homeostasis in cancer cells and the tumor microenvironment [117]. ROS may exert cytotoxic effects on cancer cells, leading to malignant cell death, thereby limiting cancer progression [118,119]. A high level of ROS may provide avenues for cancer therapy by activating various cell death pathways, such as necrosis, apoptosis, autophagy and ferroptosis; therefore, increasing ROS is one of the main anticancer strategies [120,121]. Some studies revealed that curcumin could induce excessive ROS generation, then induce oxidative stress in cancer cells. A study showed that curcumin promoted ROS production in cervical cancer Siha cells [91]. In another study, the ROS levels were elevated in gastric cancer MGC-803 cells after treatment with curcumin, suggesting that curcumin had a pro-oxidative effect [66]. Treatment with curcumin also increased ROS production in colorectal cancer SW480 cells [52]. Additionally, curcumin treatment could enhance ROS levels in breast cancer MDA-MB-231 cells [24]. Curcumin-induced ROS upregulation also triggered endoplasmic reticulum stress in prostate cancer-associated fibroblasts via the PERK-eIF2α-ATF4 axis, ultimately leading to apoptosis [74].

### 2.7. Effects on Gut Microbiota

Gut microbiota could play a vital role in health and diseases [122]. Gut dysbiosis may lead to cancer development, such as colon, gastric and breast cancers [123,124]. There are several strategies that can be used to target gut microbiota to prevent or treat cancer, such as dietary interventions, fecal microbiome transplant and targeted antibiotic approaches [125]. The studies also showed that some natural products could be anticancer, via targeting gut microbiota [126]. Curcumin significantly altered the gut microbiota composition in the H22 mice xenograft liver tumor model, and the abundances of *Bifidobacterium* and *Lactobacillus* were elevated. The oral bioavailability of curcumin was enhanced by increasing abundance of *Escherichia_shigella* [81]. Zinc complexes of curcumin attenuated degradation of intestinal mucus barrier and gut dysbiosis in a rat hepatocellular carcinoma model, and enhanced chemosensitizer for doxorubicin via gut microbiota. The ratio of *Firmicutes/Bacteroidetes* was reduced [82]. Moreover, curcumin could reduce the tumor burden in AOM-treated Il10^−/−^ mice through increasing the relative abundance of *Lactobacillales* and decreasing the relative abundance of *Coriobacterales* [127]. In the future, more studies are necessary to evaluate the effect of curcumin on various cancers via targeting gut microbiota. 

### 2.8. Adjuvant Therapy for Cancers

The biggest obstacle in targeting cancer therapy is the inevitable emergence of drug resistance in the early or late stages of drug treatment, which is a major clinical problem [128]. Clinical resistance can lead to treatment failure and eventual patient death [129]. Therefore, curcumin has been used as a promising adjuvant to improve the efficacy of many chemotherapeutic drugs. For example, incubation of curcumin with anticancer drugs such as cisplatin, doxorubicin or methotrexate, respectively, significantly reduced the IC_50_ of anticancer drugs and sensitized liver cancer HepG2 cells to anticancer drugs [80]. In addition, the combination of curcumin and metformin may have a synergistic effect, inhibiting the proliferation, migration and invasion of gastric cancer AGS cells [130]. It has also been reported that the combination of curcumin and 3′,4′-didemethylnobiletin induced cell apoptosis and cell cycle arrest of colon cancer HCT-116 cells more effectively than individual compounds [131]. In another study, in vitro and in vivo experiments demonstrated that curcumin reduced oxaliplatin resistance in colorectal cancer by inhibiting transforming growth factor beta (TGF-β)/SMAD family member 2/3 (Smad2/3) signaling [59]. In addition, curcumin combined with photodynamic therapy has better anticancer activity for several cancers, such as oral, kidney, breast, prostate, bladder and cervical cancer, and the possible mechanism is through increasing ROS generation and inducing apoptosis [132].

### 2.9. Other Mechanisms

In addition, curcumin has other anticancer mechanisms. It was demonstrated that the total iron content of breast cancer MCF-7 and MDA-MB-231 cells was enhanced after treatment with curcumin, indicating that curcumin triggered ferroptosis [30]. Another study found that curcumin decreased mitochondrial transmembrane potential and increased phosphor-γH2AX (Ser139) of gastric cancer MGC-803 cells, which indicated that curcumin could trigger mitochondrial damage and DNA damage [66]. Additionally, curcumin suppressed the inflammatory response by inhibiting the toll-like receptor 4 (TLR4)/nuclear factor kappa-B (NF-κB) signaling pathway, decreasing the expression of TLR4, myeloid differentiation primary response 88 (MyD88), NF-κB, TNF-α, interleukin-6 (IL-6), interleukin-1β (IL-1β), prostaglandin E2 (PGE2) and cyclooxygenase-2 (COX-2) in liver cancer. Meanwhile, it also inhibited tumor angiogenesis via downregulating the expression levels of vascular endothelial growth factor (VEGF), CD31 and αSMC [79].

## 3. Results from Clinical Trials

Several clinical trials have been conducted to assess the effects of curcumin on cancers (Table 2). For instance, a quasi-experimental design recruited 40 cervical carcinoma stage IIB-IIIB patients to ingest curcumin (4 g/day, 20 persons) or placebo (20 persons) for 7 days, who also received radiation therapy simultaneously. The results revealed that intake of curcumin decreased the level of the anti-apoptotic protein survivin in 15 patients (75%), and increased the level of survivin in five (25%). On the other hand, eight patients (40%) in the placebo group decreased the level of survivin, and 12 patients (60%) increased the level of survivin. The result indicated that curcumin was an effective radiosensitizer in the treatment of cervical cancer patients [133]. Moreover, 150 women participants with advanced and metastatic breast cancer received intravenous administration of curcumin (300 mg/week) + paclitaxel (80 mg/m^2^ body surface area) or placebo + paclitaxel (80 mg/m^2^ body surface area) for 12 weeks. The result showed that curcumin improved objective response rates and patient self-assessed performance status, and meanwhile reduced fatigue and did not decrease quality of life [134]. Besides, in 97 prostate cancer patients daily ingested with 1.44 g curcumin for 6–36 months, the elevation of prostate-specific antigen was suppressed during the curcumin administration period [135]. However, curcumin showed no significant effect in some cases. For example, a randomized controlled trial showed that no significant efficacy was observed with nanocurcumin supplementation (120 mg/day) in prostate cancer patients treated with radiation [136]. Additionally, treatment with curcumin (6 g/d) for 6 weeks had no significant benefits in metastatic castration-resistant prostate cancer [137]. The inconsistent results could be due to the intricate factors involved in clinical trials, and further research is necessary.

## 4. Enhancing Curcumin Bioavailability

Curcumin has shown anticancer activities. However, some limiting factors, such as its poor water solubility and extremely low oral bioavailability, could reduce its therapeutic effects [143]. Many techniques have been developed and applied to overcome this limitation [144]. For instance, protein/polysaccharide-decorated folate as a targeted nanocarrier of curcumin (fCs-Alg@CCasNPs) prolonged the sustained release of curcumin, and improved the bioavailability of curcumin, and in vivo and in vitro experiments demonstrated that fCs-Alg@CCasNPs had a higher therapeutic effect than treatment with free curcumin on pancreatic cancer and Ehrlich carcinoma [145]. Besides, a novel nano-system MSN_CurNQ was formed by loading curcumin and naphthoquinone (NQ) into the pores of mesoporous silica nanoparticles (MSN), aiming to increase the drug delivery of CurNQ via the enhanced permeation and retention effect and sustained release. The results of cellular experiments showed that MSN_CurNQ had tumor-specific toxicity and reduced the viability of cancer cells to a greater extent compared to healthy fibroblast cell lines [146]. Curcumin-loaded Gemini surfactant nanoparticles also significantly enhanced the solubility, uptake and cytotoxicity of curcumin, and inhibited breast cancer MCF-7, SkBr-3 and MDA-MB-231 cell proliferation by inducing apoptosis after effective delivery of curcumin [147]. Moreover, hydrophilic hyaluronic acid (HA) conjugated with hydrophobic curcumin form amphiphilic HA-ADH-CUR conjugates, and then subsequently self-assembled in aqueous solution to form nanoparticles HA@CUR NPs, effectively accumulated at the tumor site through endocytosis and attained a superior therapeutic effect of tumor growth inhibition [148]. Furthermore, loading curcumin onto the non-spherical delivery system zinc oxide-β cyclodextrin 3-mercaptopropionic acid (ZnO-βCD-MPA) conjugated folic acid to generate a ZnO-βCD-MPA-FA-curcumin formulation for aqueous delivery of curcumin, which allowed for sustained release of curcumin to enhance its targeting, bioavailability and release profile. Compared to free CUR, this formulation had a stronger anticancer effect on the breast cancer MDA-MB-231 cells via inducing apoptosis and had no cytotoxic effect on HEK293 normal cells [149]. In addition, curcumin–cyclodextrin/cellulose nanocrystal nano complexes were more soluble in water than free curcumin and had stronger cytotoxic activity against prostate cancer PC-3 and DU145 cells and colon cancer HT29 cells [150].

## 5. Safety of Curcumin

Curcumin has been permitted by the U.S. Food and Drug Administration as “generally regarded as safe”, and 180 mg/day of curcumin supplementation is reasonable [151,152]. Some studies revealed that curcumin showed no toxic effects in humans, and was safe and tolerable [153]. However, some adverse effects of curcumin have been observed. For example, a phase I clinical trial of oral curcumin found that curcumin was well tolerated, but diarrhea was observed in some patients [154]. Another study showed that curcumin was a safe and tolerable adjunct, but nausea was observed in some patients [138]. In addition, curcumin patients group had urinary frequency [135].

## 6. Conclusions and Perspectives

Cancer is a serious public health problem. Many studies have reported the effectiveness of curcumin in the prevention and management of various cancers, such as thyroid, breast, gastric, colorectal, liver, pancreatic, prostate and lung cancers. The potential mechanisms include inhibiting cancer cell proliferation, suppressing invasion and migration, promoting cell apoptosis, inducing autophagy, decreasing cancer stemness, increasing reactive oxygen species production, reducing inflammation, triggering ferroptosis, regulating gut microbiota, and adjuvant therapy. Meanwhile, several nanomaterials have been developed to prolong the release or targeted delivery of curcumin to cancer tissues, and further enhance the bioavailability and anticancer activities of curcumin. Moreover, the studies have shown that curcumin is generally safe and well tolerated, although some side effects have been observed, such as diarrhea and nausea. In the future, the anticancer activities of curcumin on more cancers should be evaluated, and the relative mechanisms should be explored. In addition, more methods should be studied to improve the bioavailability of curcumin in order to increase its anticancer activities. Furthermore, more clinical trials should be carried out to assess the anticancer effects of curcumin on human beings. This paper will be helpful for research and development of the third-generation function food containing curcumin.

## Figures and Tables

**Table 1:** The mechanisms of curcumin on cancers.

Study Type	Models	Dose & Duration	Effects	Mechanisms	Ref.
**Breast cancer**					
In vitro In vivo	MDA-MB-231 and MDA-MB-468 cells; female *BALB/c-nu/nu* mice with MDA-MB-231 adherent cells	10, 15, 20, 25, 30 and 35 µM, 24, 48 and 72 h	Inhibiting proliferation, invasion and migration, EMT and stemness	↓PTCH1, SMO, Gli1, Gli2, N-cadherin, vimentin, Oct4, Sox2	[27]
In vitro	MCF-7 and MDA-MB-231 cells	6.25, 25 and 100 µM, 24 h	Cytotoxicity and photosensitizing effect	↓PTP1B; ↑ROS	[28]
In vitro	MCF-7/TAMR cells	5, 10, 20, 30 and 40 µM, 48 h	Preventing cell migration and invasion, and EMT	↓N-cadherin, H19; ↑E-cadherin	[29]
In vitro	MCF-7 and MDA-MB-231 cells	5, 10, 20, 40, 60, 80, 100, 120 and140 μM, 24 and 48 h	Inhibiting cell viability; Promoting oxidative stress, ER stress, and ferroptosis	↑HO-1, Nrf2, ROS, HSPA5, ATF4, DDIT3, MDA, FTL, TFRC, FTH1, BACH1, RELA, USF1, NFE2L2; ↓GPX4, GSH	[30]
In vitro In vivo	MDA-MB-231 cell; BALB/ c nude mice with MDA-MB-231 cells	5, 10, 20 and 50 μM, 24 h; 25 g/kg, 4 weeks	Inhibiting cell proliferation and cancer growth	↑GFPu, miR-142-3p; ↓PSMB5, PSMB1, P300, CT-1	[31]
In vitro, In vivo	MCF-7, MDA-MB-231 and MDA-MB-468 cells; female BALB/c nude mice with MDA-MB-231 cells	20 and 40 µM, 48 h; 100 mg/kg/2 days, 21 days	Inhibiting proliferation, migration and invasion; Promoting apoptosis; Blocking the cell cycle	↓cyclin A1, CDK1, Bcl-2, EZH2; ↑Caspase-9, DLC1	[32]
In vitro	MCF-7 and MDA-MB-231 cells	10, 15, 20, 25, 30, 35 and 40 µM, 24 and 48 h	Inhibiting cell viability, invasion and migration, mammosphere formation and differentiation abilities, stem cell properties	↓CD44^+^CD24^−^ subpopulation, vimentin, fibronectin, β-catenin, Oct4, Nanog, Sox2; ↑E-cadherin	[33]
In vitro	HCC-38, UACC-3199, and T47D cells	5 and 10 µM, 3 days	Suppressing proliferation and methylation	↓DNMT1, miR-29b, SNCG; ↑BRCA1, TET1, DNMT3	[34]
In vitro	MCF-7 and MDA-MB-231 cells	5, 10 and 25 µM, 48 h	Inhibiting cell vitality; Inducing apoptosis	↓TLR4, TRIF, IRF3, IFN-α/β	[35]
In vitro	MCF-7, MDA-MB-453 and MDA-MB-231 cells	5, 10, 15, 20, 25 and 30 µM, 24, 48 and 72 h	Inhibiting proliferation, invasion and metastasis; Inducing apoptotic cell death and cell cycle arrest	↓Src, pSTAT-1, p-Akt, p-p44/42, Ras, c-raf, vimentin, β-catenin, p53, Rb, p-Rb, Bax, Bcl-2, Bcl-xL, Mcl-1; ↑PIAS-3, SOCS-1, SOCS-3, ROS, NF-κB, PAO, SSAT, p21, Bak	[36]
In vitro	T47D, MCF7, MDA-MB-415, SK-BR-3, MDA-MB-231, MDA-MB-468 and BT-20 cells	10 and 30 µM, 24 and 48 h	Inhibiting proliferation; Inducing G2/M arrest and apoptosis	↓CDC25, CDC2, p-Akt, p-mTOR, p-S6, Bcl-2; ↑p21, Bax, Cleaved-caspase-3	[37]
In vitro	MDA-MB-231 and CAL-51 cells	5 µM, 48 h	Inhibiting proliferation; Inducing apoptosis	↓Bcl-2, RAD51; ↑ROS, Bax, γH2AX	[24]
**Lung cancer**					
In vitro In vivo	H1650, H1299, H460 and A549 cells; BALB/c nude mice with A549 cells	10, 20 and 40 μM, 24 h; 50 mg/kg, 22 days	Accelerating apoptosis; Inhibiting migration, invasion and xenograft tumor growth	↓circ-PRKCA, ITGB1; ↑miR-384	[38]
In vitro In vivo	H460, H1299, H1975, A549, SCC-827, PC-9 and CMT-64 cells; female C57bl/6j mice with CMT-64 cells	4, 8, 12, 16, 20, 24 and 28 μg/mL, 24 h; 5 mg/kg, 24 h	Inhibiting of tumor growth and volume; Ameliorating the immunosuppressive micro-environment	↓MDSCs cells, Treg cells, IL-10; ↑NK cells	[39]
In vitro	H1299 and A549 cells	2.5, 5 and 7.5 μM, 48 h	Decreasing migration, invasion and EMT Process	↑TAp63α, E-cadherin, ZO-1; ↓Vimentin, N-cadherin, miR-19a, miR-19b	[40]
In vitro In vivo	A549 and H1299 cells; female C57BL/6 mice with Lewis lung carcinomas cells	5, 10, 20, 30 and 40 μM, 24 h; 100 mg/kg/day, 15days	Inhibiting tumor growth; Inducing ferroptosis and autophagy	↓SOD, GSH, SLC7A11, GPX4, p62; ↑MDA, iron, ACSL4, Beclin1, LC3-II, autolysosome, mitochondrial damage	[41]
In vitro In vivo	A549/GR and H520/GR cells; BALB/c nude mice with A549/GR cells	50, 100 and 150 μM, 48 h; 100 mg/kg, 3 weeks	Suppressing proliferation; Promoting apoptosis	↑lncRNA-MEG3, PTEN	[42]
In vitro	A549, NCI-H1299	5, 25, 125 and 250 nM, 24, 48 and 72 h	Suppressing sphere size and number, and stemness	↓ALDH, CD133, Epcam, Oct4, TAZ; ↑Hippo pathway, p-TAZ	[43]
In vitro	H446 cells	5, 10, 15 and 20 μM, 24 and 48 h	Inducing cell apoptosis; Regulating cell cycle	↓Bcl-2, CCNF, LOX1, MRGPRF, and VEGFB; ↑Bax, cytochrome-C, miR-548ah-5p	[44]
In vitro	A549 cells	1, 2, 5, 10 and 20 μM, 24 and 48 h	Inhibiting migration and invasion	↓E-cadherin, sE-cad, vimentin, slug; ↑N-cadherin, snail, MMP-9	[45]
In vitro	A549 cells	25, 50 and 100 μM, 48 h	Inhibiting proliferation; Inducing apoptosis	↓14-3-3 proteins, p-Bad, p-AKT/AKT, Caspase-9, PARP; ↑Cleaved-caspase-9, Cleaved-PARP	[46]
In vitro	A549 cells	5, 10, 20 and 40 μM, 24, 48, 72 and 96 h	Inhibiting proliferation; Inducing apoptosis and autophagy	↓p-Akt, p-mTOR, p62, LC3-I; ↑Beclin1, LC3-II	[47]
In vitro	A549 cells	10, 20 and 40 μM, 12, 24 and 48 h	Inhibiting migration and invasion	↓miR-25-5p; ↑miR-330-5p	[48]
In vitro	A549 and H1299 cells	0.5, 1, 5, 10 and 20 µM, 24, 48 and 72 h	Inhibiting colony formation; Promoting apoptosis and autophagy	↓p-mTOR, p-S6, p-PI3K, p-Akt ↑LC3-II/ LC3-I, Beclin-1	
**Colorectal cancer**					
In vitro In vivo	TCO1 and TCO2 cells; SCID mice with organoid cells	0.6, 2, 6 and 20 µg/mL, 72 h; 20 mg/day, 21 days	Inducing necrotic lesions and apoptosis; Inhibiting stemness and proliferation	↓cyclin D1, c-MYC, p-ERK, CD44, CD133, LGR5	[49]
In vitro In vivo	CC531 cells; tumor-bearing rats with CC531 cells	15, 20, 25 and 30 µM, 24, 48 and 72 h; 200 mg/kg/day, 28 days	Reducing proliferation and migration; Diminishing global tumor progression	↑AST, ALP, albumin; ↓cholinesterase, cholesterol, and total protein	[50]
In vitro	SW620 cells	1, 5 and 25 μM, 48 h	Inhibiting tumor sphere formation; Inducing apoptosis and autophagy	↓GP1BB, COL9A3, COMP, AGRN, ITGB4, LAMA5, COL2A1, ITGB6, LGR5, TFAP2A, ECM; ↑Autolysosomes, autophagosomes	[51]
In vitro In vivo	SW480 and HT-29 cells; BALB/c nude mice with SW480 cells	10, 20, 30, 40, 50 and 60 µM, 24 h; 100 mg/kg/day, 3 weeks	Inhibiting proliferation and tumor volume and weight; Inducing apoptosis	↓NNMT, p-STAT3, G2/M phase cell cycle arrest; ↑ROS	[52]
In vitro	HCT-116/L-OHP cells	10, 20, 30 and 40 µM, 48 h	Inhibiting proliferation, migration and invasion; Arresting cell cycle distribution	↓ERCC1, Bcl-2, GST-π, MRP, P-gp; ↑miR-409-3p	[53]
In vitro	5-FU resistant HCT-116 cells	5, 10, 20 and 40 μM, 48 h	Inhibiting proliferation; Inducing apoptosis; Blocking G0/G1 phase	↓E-cadherin, β-catenin, TCF4, Axin; ↑TET1, NKD2, vimentin	[54]
In vitro	SW480 cells	0.1, 0.2 and 0.4 µM, 24 h	Inhibiting EMT and the expression of DNMTs	↑E-cadherin; ↓N-cadherin, twist, snail, vimentin, CDX2, DNMT1, DNMT3a, Wnt3a, β-catenin	[55]
In vitro In vivo	HCT8 and HCT8/DDP cells; Nude mice with HCT8/DDP cells	10 μM, 48 h; 1 g/kg/week, 42 days	Reducing tumor volume and weight; Promoting apoptosis	↓Bcl-2, KCNQ1OT1; ↑cytochrome C, Bax, Cleaved-caspase-3, Cleaved-PARP1, miR-497	[56]
In vitro	HCT116, HCT8, SW480 and SW620 cells	10 μM, 24 h	Reducing clone formation	↑NBR2, p-AMPK, p-ACC; ↓p-S6K/p-S6, Mtor, S-phase	[57]
In vitro	SW480 and 5FU-SW480 cells	5, 10, 15, 20, 25, 30, and 50 μM, 48 and 72 h	Inducing apoptosis; Decreasing colony formation and migration	↓insulin, IGF-1 receptors	[58]
In vitro, In vivo	HCT116/OXA and HCT116 cells; BALB/c nude mice with HCT116/OXA cells	1, 2, 4, 8, 16, 32 and 64 μM, 48 h; 60 mg/kg, 3 weeks	Inhibiting tumor volumes and weights; Decreasing the migratory ability	↓p-p65, Bcl-2, p-Smad2, p-Smad3, N-cadherin, TGF-β; ↑Cleaved-caspase3, E-cadherin	[59]
In vitro	HT-29 and DLD-1 cells	15, 20 and 25 μM, 48 h	Inducing apoptosis and G2/M cell cycle arrest	↓p-Akt, p-Bad, Bcl-2, GPX1, GPX4; ↑ROS, HSP27, Bad, cPARP, Beclin 1, p62	[60]
In vitro In vivo	SW480 cells; female nude mice with SW480 cells	40 μM, 24 h; 200 mg/kg, 5 days	Suppressing proliferation	↓β-catenin, TCF4, miR-21, miR-130a; ↑Nkd2	[61]
In vitro	HCT-116 and HCT-8 cells	2.5, 5, 10, 20 and 40 µM, 24 h	Inhibiting proliferation, migration and stem-cell like characteristics	↑CD44	[62]
**Head and Neck Cancer**					
In vitro In vivo	HNSCC cell lines SNU1076, SNU1041, FaDu and SCC15; C57BL/6 mice with SCC15 cells	1, 2, 5, 10, 20, 40 and 80 µM, 1, 3, 6, 12 and 24 h; 50 mg/kg, 6 weeks	Inhibiting cell viability, invasion, EMT, and tumor formation and growth; Enhancing ability of effector T cells to kill cancer cells and immune response to tumors	↓p-STAT3, TIM-3^+^CD4^+^ T cells, PD-1^+^CD8^+^ T cells, TIM-3^+^CD8^+^ T cells, CD4^+^CD25^+^FoxP3^+^ Treg cells, PD-1, TIM-3; ↑E-cadherin, CD8^+^ T cells, IFN-γ	[63]
In vitro	SCC-9, FaDu and HaCaT cells	50, 25, 10, 5, 2.5, 1.25 and 0.75 μM, 24 and 48 h	Reducing cell viability; Inducing cell cycle arrest; Modifying cytoskeleton organization	↓procaspase-3, EGFR, PLD1, RPS6KA1, p-mTOR, p-AKT, PI3K; ↑Caspase-3, PRKCG, EGF	[64]
**Gastric cancer**					
In vitro	AGS cells	10, 20, 30, 40, 50, 60, 70, 80, 90 and 100 µM, 24, 48 and 72 h; 50 mg/kg, 6weeks	Inducing apoptosis; Suppressing proliferation	↓Bcl-2, survivin; ↑Bax, the proportion of Sub-G1 cells	[65]
In vitro	MGC-803 cells	5, 10, 15, 20, 40 and 60 μM, 24, 48 and 72 h	Inhibiting proliferation and migration; Promoting mitochondrial and DNA damage, and apoptosis	↓Δ ψm, cyclin E1, DNMT1, p-Rb, methylated CpG sites; ↑ROS, ATM, ATR, GADD45A, p21, p-p53, p-γH2AX	[66]
In vitro	SGC-7901 cells	10, 20, 40 and 80 µM, 48 h	Suppressing proliferation, invasion, and cytoskeletal remodeling ability; Inducing apoptosis	↓Gli1, Foxm1, β-catenin, pseudopods, skeleton fibers, vimentin; ↑S stage, E-cadherin	[67]
In vitro In vivo	SGC-7901 cells; BALB/c male nude mice with SGC-7901 cells	50 μM, 24, 48 and 96 h	Decreasing migration, invasion and growth of transplanted tumors; Promoting cell apoptosis	↓Bcl-2, cyclin D1, CDK4; ↑miR-34a	[68]
In vitro	SGC-7901 and BGC-823 cells	10, 20 and 40 μM, 24 h	Inhibiting proliferation; Promoting apoptosis and autophagy	↓Bcl-2, Bcl-xL, LC3I, PI3K, p-Akt, p-mTOR; ↑Bax, Beclin1, ATG3, Cleaved-caspase-3, Cleaved-PARP, ATG5, LC3II, p53, p21	[69]
In vitro In vivo	SGC-7901 cells; Balc/c nude mice with SGC7901 cells	25 μM, 3, 5 and 7 days; 100 mg/kg, 2 weeks	Inhibiting proliferation, gastrin and gastric acid secretion; Promoting apoptosis	↑Caspase-3	[70]
**Bladder cancer**					
In vitro	T24 and RT4 cells	10, 15, 20 and 25 µM, 48 and 72 h	Inhibiting cell growth, migration and invasion; Inducing cell cycle arrest	↓Trop2, cyclin E1; ↑G2/M cell populations, p27	[71]
In vitro	J82, TCCSUP and T24 cells	1, 5, 10 and 20 µM, 24, 48 and 72 h	Decreasing invasion and tumorigenicity; Increasing apoptosis	↓miR-7641; ↑p16	[72]
**Prostate Cancer**					
In vitro	PC-3 and DU145 cells	10, 20, 30, 40 and 50 µM, 12, 24 and 48 h	Reducing cell viability, migration and invasion; Promoting apoptosis	↓PCLAF, Bcl-2, Caspase-3; ↑miR-30a-5p, Bax, Cleaved-caspase-3	[73]
In vitro	Prostate-CAFs, PC-3 and NAFs cells	10, 20 and 30 μM, 8, 12 and 24 h	Inducing apoptosis and ER stress; Regulating cell cycle	↓Bcl-2, ΔΨm; ↑Cleaved-caspase-3, Bax, Bims, Cleaved-PARP, Puma, p-p53, ROS, p-ERK, p-eIF2α, CHOP, ATF4	[74]
In vitro In vivo	LNCaP and 22Rv1 cells; male TRAMP mice	5, 25 and 50 μM, 24, 48 and 72 h; 200 mg/kg/day, 30days	Inhibiting growth; Inducing apoptosis	↓CYP11A1, HSD3B2, StAR, testosterone, dihydrotestosterone; ↑AKR1C2, SRD5A1, CYP17A1	[75]
In vitro	22RV1, PC-3 and DU145 cells	1, 5, 10 and 20 μM, 4 days	Suppressing proliferation	↓cyclin D1, PCNA, β-catenin, c-MYC; ↑p21, miR-34a	[76]
**Thyroid cancer**					
In vitro	K1, FTC-133, BCPAP and 8505C cells	10, 12.5, 20, 25, 30, 40 and 50 µM, 24 and 72 h	Inhibiting cell growth; Inducing autophagy	↑LC3-II, Beclin-1, p-p38, p-JNK, p-ERK1/2; ↓p62, p-PDK1, p-Akt, p-p70S6, p-p85S6, p-S6, p-4E-BP1	[77]
In vitro	TPC-1 and BCPAP-R cells	2.5, 5, 10, 20 and 40 µM, 24 h	Inhibiting cell viability, invasion, migration and EMT	↓MMP-9, MMP-2, N-cadherin, vimentin, fibronectin, p-JAK, p-JAK2, p-JAK3, p-STAT1, p-STAT2; ↑E-cadherin, miR-301a-3p	[78]
**Liver cancer**					
In vitro In vivo	HepG2, Huh-7 and MHCC-97H cells; BALB/c-nu nude mice with HepG2 cells	1.2, 2.4, 4.8 and 9.6 µg/mL, 24 and 48 h; 120 and 240 mg/kg/day, 15 days	Reducing tumor volume and weight, and angiogenesis	↓MDSCs, GM-CSF, G-CSF, TLR4, MyD88, p-IKKα, p-IKKβ, NF-κB, TNF-α, IL-6, IL-1β, PGE2, COX-2, VEGF, CD31, α-smooth	[79]
In vitro	HepG2 and HuT78 cells	5 and 10 μM, 24 h	Inducing cell death	↓lactate, ldh-a, mct-1, mdr-1, stat-3, HIF-1α, HCAR-1; ↑NO	[80]
In vitro	HepG2 cells	20, 50, 80 and 100 μM, 24, 48 and 72 h	Inhibiting proliferation, migration and invasion; Promoting apoptosis	↓HSP70, eHSP70, TLR4	[25]
In vitro In vivo	Bel-7,402 and HepG2 cells; male BALB/c mice with H22 cells	15 and 30 μM, 24, 48 and 72 h; 100 mg/kg/day, 14 days	Inducing apoptosis, G2/M cell cycle arrest; Modulating gut microbiota	↓p-PI3K, p-Akt, p-mTOR, tumors weights and sizes; ↑Cleaved-caspase-3, *Lactobacillus*, Epsilonbacteraeota, Helicobacterac-eae, Campylobacterales, *Helicobacter*, *Escherichia-shigella*, *Bifidobacterium*, *Campylobacteria*	[81]
In vitro In vivo	HepG2 and SK-HEP1 cells; male BALB/c mice H22 and HepG2 cells	20, 40, 60, 80, 100, 120 and 140 nM, 24 h; 100 mg/kg curcumin or Zn (II)-curcumin, 2 weeks	Inhibiting tumor growth; Regulating gut microbiota; Improving intestinal permeability	↓*Firmicutes*, *unclassified Lachnospiraceae*, *Clostridium cluster XIVa*, *Pseudoflavonifractor*, *Oscillibacter*; ↑*Bacteroidetes*, *Barnesiella*, *Unclassified_Porphyromonadaceae*, *Paraprevotella*, *Prevotella*, zonula occludens-1, occludin	[82]
**Ovarian cancer**					
In vitro	SKOV3 cells	10, 20, 30, 40 and 50 μM, 6, 12 and 24 h	Inhibiting migration and invasion	↓STAT3, fascin	[83]
In vitro	SKOV3 cells	20 μM, 96 h	Inhibiting cell migration and EMT	↓DNMT3a, β-catenin, cyclin D1, c-Myc, fibronectin, vimentin; ↑SFRP5, E-cadherin	[84]
In vitro	SK-OV-3 and A2780 cells	5, 10, 20, 40 and 80 μM, 24, 48 and 72 h	Inducing apoptosis and autophagy	↓p62, p-AKT, p-mTOR, p-p70S6K; ↑Caspase-9, PARP, Atg3, Beclin-1, LC3B-I/II	[85]
In vitro In vivo	SKOV3 and A2780 cells; BALB/c athymic mice with A2780 cells	10, 20 and 40 μM, 24, 48 and 72 h; 15 mg/kg/2days, 5 weeks	Inhibiting proliferation; Promoting apoptosis	↓PCNA, miR-320a; ↑Bax, Cleaved-caspase-3, circ-PLEKHM3, SMG1	[86]
**Oral Cancer**					
In vitro	HSC-4 and Ca9-22 cells	15 μM, 48 h	Decreasing invasion, migration and EMT	↓vimentin, p-c-Met, p- ERK, pro-MMP9; ↑E-cadherin	[87]
**Pancreatic Cancer**					
In vitro	Panc-1 and MiaPaCa-2 cells	6, 10 and 12 µM, 24 h	Reducing cell survival; Inducing apoptosis and DNA damage	↓G0/G1-fraction; ↑yH2AX-MFI, G2/M-fraction, S-phase cells	[88]
In vitro	PANC-1 cells	2.5, 5, 10 and 20 µM, 72 h	Inducing apoptosis	↑Cleaved-caspase-3, miR-340, Cleaved-PARP; ↓PARP, XIAP	[89]
In vitro	Patu8988 and Panc-1 cells	5, 10, 15 and 20 μM, 48 and 72 h	Inhibiting migration and invasion; Inducing apoptosis	↓NEDD4, p-Akt, p-mTOR; ↑PTEN, p73, β-TRCP	[90]
**Cervical Cancer**					
In vitro	Siha cells	5, 15, 30 and 50 µM, 6, 12, 24 and 48 h	Inhibiting proliferation; Inducing G2/M cell cycle arrest, apoptosis, autophagy	↓cyclins B1, cdc25; ↑ROS, p62, LC3I/II, Cleaved-caspase-3, Cleaved-PARP, p53, p21	[91]
In vitro	Siha cells	20 µM, 72 h	Decreasing EMT and migration	↓N-cadherin, vimentin, slug, Zeb1, PIR, pirin; ↑E-cadherin	[92]
**Tongue Cancer**					
In vitro	CAL 27 cells	10, 25, 50 and 100 µM, 16 and 24 h	Inhibiting proliferation and migration; Promoting apoptosis and S-phase cell cycle arrest	↓Bcl-2; ↑Bax, Cleaved-caspase-3, S-phase cells	[93]
**Brain Cancer**					
In vitro	SNB19 and A1207 cells	10, 15, 20 and 25 µM, 48 and 72 h	Suppressing proliferation, migration and invasion; Inducing apoptosis and cell cycle arrest	↓NEDD4, Notch1, p-Akt; ↑G2/M phase	[94]

Abbreviations: ACSL4, acyl-CoA synthetase long-chain family member 4; Akt, protein kinase B; AKR1C2, Aldo-Keto reductase 1C2; ALP, alkaline phosphatase; AST, aspartate transaminase; ATF4, activating transcription factor 4; Atg3, autophagy related 3; Atg5, autophagy related 5; Bax, Bcl-2 associated X protein; BACH, BTB domain and CNC homolog 1; Bcl-2, B-cell lymphoma-2; Bim, Bcl-2 interacting mediator of cell death; Bcl-xL, B-cell lymphoma-extra-large; Caspase-3, cysteinyl aspartate specific proteinase 3; CDK1, cyclin dependent kinase 1; CDK4, cyclin dependent kinase 4; CDX2, caudal type homeobox 2; CHOP, C/EBP homologous protein; COX-2, cyclooxygenase-2; CYP11A1, Cytochrome P450scc; HSD3B2, type 2 3β-hydroxysteroid dehydrogenase; CYP17A1, Cytochrome P450(17α); DDIT3, DNA damage inducible transcript 3; DLC1, deleted in liver cancer 1; DNMT1, DNA methyltransferase 1; DNMT3a, DNA Methyltransferase 3 Alpha; ECM, extracellular matrix; ERCC1, excision repair cross-complementing gene; EGFR, phospho-epidermal growth factor receptor; eHSP70, extracellular HSP70; eIF2α, eukaryotic translation initiation factor-2α; EMT, Epithelial-mesenchymal transition; Epcam, epithelial cell adhesion molecule; ER stress, endoplasmic reticulum stress; ERK, extracellular regulated protein kinases; FTH1, ferritin heavy chain 1; G-CSF, granulocyte-colony stimulating factor; GFPu, a short degron CL1 fused to the COOH-terminus of green fluorescent protein; GM-CSF colony-stimulating factor; Gli1, Glioma-associated oncogene family zinc finger 1; Gli2, Glioma-associated oncogene family zinc finger 2; GPX4, glutathione peroxidase 4; GSH, glutathione; HO-1, hemeoxygenase-1; HSP70, heat shock protein 70; GST-π, glutathione thio-transferase π; HSPA5, heat shock 70 kDa protein 5; IL-1β, interleukin-1β; IL-6, interleukin-6; IKK, inhibitor of nuclear factor kappa-B kinase; JAK, Janus kinase; ITGB1, integrin beta 1; JNK, c-Jun N-terminal kinase; LC3, microtubule-associated protein light chain 3; MDA, malondialdehyde; MDSCs, myeloid-derived suppressor cells; MMP-2, matrix metalloprotein-2; MMP-9, matrix metalloprotein-9; MRP, multidrug resistance-related protein; mTOR, mammalian target of rapamycin; MyD88, myeloid differentiation primary response 88; Nanog, Nanog Homeobox; NEDD4, neural precursor cell expressed developmentally down-regulated protein 4; NFE2L2, NFE2-related factor 2; NNMT, Nicotinamide N-Methyltransferase; NF-κB, nuclear factor kappa-B; Nrf2, nuclear factor-erythroid 2-related factor-2; Oct4, Octamer-binding transcription factor 4; PARK7, Parkinson’s disease protein 7; P300, histone acetyltransferase p300; p38 MAPK, p38 mitogen-activated protein kinase; PARP, poly (ADP-ribose) polymerase; PCLAF, PCNA clamp associated factor; PD-1, Programmed cell death protein 1; PD-L1, Programmed death-ligand 1; PGE2, prostaglandin E2; PI3K, Phosphatidylinositol-3-kinase; P-gp, P-glycoprotein; PSMB, proteasome 20S subunit beta; PTEN, phosphatase and tensin homolog; PTP1B, Protein tyrosine phosphatase 1B; PTEN, Phosphatase and tensin homolog deleted on chromosome 10; PTCH1, Patched; PUMA, p53 upregulated modulator of apoptosis; RELA, v-rel reticulo-endotheliosis viral oncogene homolog A; ROS, Reactive oxygen species; sE-cad, soluble E-cadherin; SFRP5, secreted frizzled-related protein 5 gene; Smad2/3, SMAD family member 2/3; SMG1, suppressor of morphogenesis in genitalia 1; SMO, Smoothened; SOD, superoxide dismutase; Sox2, Sex determining region Y-box 2; SRD5A1, steroid 5α-reductase type 1; STAT, signal transducer and activator of transcription; StAR, steroidogenic acute regulatory protein; STAT3, signal transducer and activator of transcription 3; TCF4, transcription factor 4; TET1, tet methyl-cytosine dioxygenase 1; TGF-β, transforming growth factor beta; TIM-3, T-cell immunoglobulin and mucin-domain 3; TLR4, toll-like receptor 4; TNF-α, tumor necrosis factor α; Tregs, Regulatory T cells; TRAMP, the transgenic adenocarcinoma of the mouse prostate; USF1, upstream transcription factor 1;VEGF, vascular endothelial growth factor; Wnt3a, Wnt family member 3a; XIAP, X-linked inhibitor of apoptosis; Zeb1, Zinc finger E-box binding homeobox 1; ZO-1, zonula occludens-1; ΔΨm, mitochondrial membrane potential.

**Table 2:** The effects of curcumin on cancers from clinical trials.

Therapy	Study Type	Subjects	Administration Methods	Dose & Duration	Outcomes	Ref.
**Cervical Cancer**						
Curcumin + radiation	Quasi-experiment	40 advanced cervical cancer patients	Oral administration	4 g/day, 7 days	Lowering survivin levels	[133]
**Breast Cancer**						
Curcumin + paclitaxel	RCT	150 women with metastatic and advanced breast cancer	Intravenous administration	300 mg/week (curcumin), 12 weeks; 80 mg/m^2^ body surface area/week (paclitaxel), once a week for 12 consecutive weeks	Improving objective response rate and patient self-assessed overall performance status	[134]
**Colorectal Cancer**						
Curcumin C3 complex + standard-of-care chemotherapy (FOLFOX ± bevacizumab)	Open-labelled RCT	27 patients with stage IV disease metastatic colorectal cancer, aged >18 y	Oral administration	2 g curcumin C3 complex/d (80% curcumin and 20% dimethoxy-curcumin and bisdemethoxycurcumin), ≤ 12 cycles of chemotherapy	Curcumin was safe and tolerable, increasing overall survival and objective response rate	[138]
Curcuminoids capsules	RCT	72 patients with stage 3 colorectal cancer, aged >20 y	Oral administration	500 mg/day, 8 weeks	Lowering serum C-reactive protein levels, enhancing functional scales and the global quality of life	[139]
**Prostate Cancer**						
Curcumin	RCT	97 prostate cancer patients	Oral administration	1440 mg/day, 6–36 months	Reducing prostate specific antigen	[135]
Nanocurcumin	RCT	64 prostate cancer patients	Oral administration	120 mg/day, 3 days before and during radiotherapy	Not efficacious	[136]
Curcumin + chemo-therapy with docetaxel	Phase II RCT	50 metastatic castration-resistant prostate cancer patients, aged >18 y	Oral administration	6 g/d (curcumin), 3 weeks; 75 mg/m^2^ body surface area (docetaxel), first day of every 3 weeks for 6 cycles	Not efficacious	[137]
**Pancreatic Cancer**						
Gemcitabine + Meriva^®^ (curcumin complexed with soy lecithin, 1:2)	Single center, single arm, prospective phase II trial	52 pancreatic cancer patients, aged >18 y	Oral administration	2000 mg (Meriva^®)^, 28 day; 10 mg/m^2^/min (gemcitabine), on days 1, 8, 15	Raising the efficiency of gemcitabine translating in a response rate	[140]
**Endometrial Cancer**						
Curcumin phytosome	Open-label, non-randomized phase II study	7 endometrial cancer patients	Oral administration	2 g/day, 2 weeks	Reducing major histocompatibility complex expression levels on leukocytes, inducible T cell costimulatory expression by CD8^+^ T cells and the frequency of monocytes, increasing CD69 levels on CD16^−^ NK cells	[141]
**Oral Cancer**						
APG-157 (including curcumin)	Phase II RCT	13 normal subjects and 12 patients with oral cancer	Oral administration	100 and 200 mg, each hour for 3 consecutive hours	Reducing inflammation, *Bacteroides* and ratio of *Firmicutes*/*Bacteroidetes*	[142]

Abbreviations: FOLFOX, folinic acid/5-fluorouracil/oxaliplatin chemotherapy; RCT, Randomized controlled trial.
